# Genomics, genetic engineering and artificial cells

**DOI:** 10.4103/0972-2327.64618

**Published:** 2010

**Authors:** Sanjeev V. Thomas

**Affiliations:** Editor, Annals of Indian Academy of Neurology, Department of Neurology, Sree Chitra Tirunal Institute for Medical Sciences and Technology, Trivandrum - 695011, Kerala, India. E-mail: editor@annalsofian.org

May 2010 was highlighted by the news of scientists creating an artificial cell. Synthetic biology is a new field that combines chemistry, computer science, molecular biology, genetics and cell biology to breed industrial life forms that can secrete fuels, vaccines or other commercial products. Several companies have already approached the Synthetic Genomic Inc. with requests to produce bacteria and algae that could mop up carbon dioxide or be a source of renewable energy. Artificial cells have numerous therapeutic potentials. Genetically engineered cells and artificial cells can deliver neuro-hormones, various neuro-peptides and other neurotransmitters to specific locations in the nervous system. They can probably replace degenerating cells in various disorders. The J Craig Ventor Institute that steered this research had announced that this was the first artificial cell completely controlled by man-made genetic instructions, which can survive and reproduce by itself. Earlier, other scientists had created artificial cells that could perform several functions. Scientists and experts in environment, ethics, philosophy and religion have received this news with mixed response. Some have considered this a turning point in the relationship between man and nature. The ethical debates and environmental concerns triggered by cloning, stem cell technology and genetic engineering will be further enhanced by the creation of artificial cells.

Health professionals, particularly neurologists, have been paying little attention to the health issues of urbanization. The World Health Organization (WHO) has chosen Urbanization and Health as the world health theme for the year 2010. Less than 30% of the population in India currently lives in urban areas. There is a strong migration of population from villages to urban areas in India as elsewhere. It is estimated that by the year 2020, this proportion would increase to 41%. The health outcomes are largely determined by environmental, social and physical infrastructure conditions. A variety of social determinants like water and sanitation, quality of air, living and working conditions, access to services and resources influence the health outcomes. Public health experts indicate that communicable diseases such as HIV/AIDS and tuberculosis, chronic diseases such as heart disease and diabetes, mental disorders, and deaths due to violence and road traffic injuries are all driven by these underlying social determinants. Unplanned urban development can lead to proliferation of slums, which in turn can lead to poor health for those living there. WHO has collaborated with central and state governments in India to promote healthy cities. WHO has collaborated with the Government of India and states to promote healthy city plans, and prepare tools and guidelines for building up healthy cities. The changing demographic patterns and increasing proportion of older persons living in congested cities are likely to alter the epidemiological patterns of stroke, CNS infections, epilepsy and several other neurological disorders. Neurologists and other neuroscientists need to be aware of the impact of rapid urbanization on health and be willing to adapt to the new situations and demands.

This issue of the journal carries several articles related to CNS infections, demyelination, stroke and epilepsy. Demyelinating diseases have close relationship to infections. Acute Gullain Barre syndrome and acute demyelinating encephalomyelitis are typical examples for that. A close association between infections and multiple sclerosis (MS) had been postulated in several epidemiological studies as well as laboratory studies. In this issue, we carry an article that discusses the relationship between MS and infections. Fungal infections of the CNS have increased considerably after the advent of HIV infection and AIDS. The diagnosis of fungal infections in immunocompetent persons is often delayed. The article on the spectrum of fungal infections in immunocompetent persons would draw the attention of the readers to suspect this condition at an early stage and initiate treatment wherever appropriate. Epilepsy continues to be a major clinical problem in India. It is estimated that there are over 1 million people with epilepsy who may benefit from epilepsy surgery. In India, at present, there are only a handful of institutions offering surgery for epilepsy and only a few hundred patients undergo surgery for epilepsy every year. The Indian Epilepsy Society has brought out a national guideline to assist the physicians to provide optimal care for persons with epilepsy. The article on epilepsy surgery in this issue is an adaptation of the section on guidelines prepared by the Indian Epilepsy Society. We hope that our esteemed readers will find these articles very helpful.

